# Evaluation of Active Brown Adipose Tissue by the Use of Hyperpolarized [1-^13^C]Pyruvate MRI in Mice

**DOI:** 10.3390/ijms19092597

**Published:** 2018-09-01

**Authors:** Mette Ji Riis-Vestergaard, Peter Breining, Steen Bønløkke Pedersen, Christoffer Laustsen, Hans Stødkilde-Jørgensen, Per Borghammer, Niels Jessen, Bjørn Richelsen

**Affiliations:** 1 Department of Internal Medicine and Endocrinology, Aarhus University Hospital, 8200 Aarhus N, Denmark; peter.breining@clin.au.dk (P.B.); amtssp@gmail.com (S.B.P.); bjrichelsen@gmail.com (B.R.); 2 Institute of Clinical Medicine, Aarhus University, 8200 Aarhus N, Denmark; 3 MR Research Center, Institute of Clinical Medicine, Aarhus University, 8200 Aarhus N, Denmark; cl@clin.au.dk (C.L.); hsj@clin.au.dk (H.S.-J.); 4 Department of Nuclear Medicine & PET Centre, Aarhus University Hospital, 8000 Aarhus C, Denmark; perborghammer@gmail.com; 5 Department of Clinical Pharmacology, Aarhus University Hospital, 8000 Aarhus C, Denmark; niels.jessen@biomed.au.dk

**Keywords:** brown adipose tissue, UCP1 expression, cold exposure, hyperpolarized pyruvate MRI, FDG PET

## Abstract

The capacity to increase energy expenditure makes brown adipose tissue (BAT) a putative target for treatment of metabolic diseases such as obesity. Presently, investigation of BAT in vivo is mainly performed by fluoro-d-glucose positron emission tomography (FDG PET)/CT. However, non-radioactive methods that add information on, for example, substrate metabolism are warranted. Thus, the aim of this study was to evaluate the potential of hyperpolarized [1-^13^C]pyruvate Magnetic Resonance Imaging (HP-MRI) to determine BAT activity in mice following chronic cold exposure. Cold (6 °C) and thermo-neutral (30 °C) acclimated mice were scanned with HP-MRI for assessment of the interscapular BAT (iBAT) activity. Comparable mice were scanned with the conventional method FDG PET/MRI. Finally, iBAT was evaluated for gene expression and protein levels of the specific thermogenic marker, uncoupling protein 1 (UCP1). Cold exposure increased the thermogenic capacity 3–4 fold (*p* < 0.05) as measured by *UCP1* gene and protein analysis. Furthermore, cold exposure as compared with thermo-neutrality increased iBAT pyruvate metabolism by 5.5-fold determined by HP-MRI which is in good agreement with the 5-fold increment in FDG uptake (*p* < 0.05) measured by FDG PET/MRI. iBAT activity is detectable in mice using HP-MRI in which potential changes in intracellular metabolism may add useful information to the conventional FDG PET studies. HP-MRI may also be a promising radiation-free tool for repetitive BAT studies in humans.

## 1. Introduction

The prevalence of obesity increases globally and conventional treatment has proven difficult with rather unsuccessful results [1,2]. Thus, there is an urgent need for alternative strategies [3,4]. One avenue of interest has been to increase energy expenditure by targeting brown adipose tissue (BAT). BAT is a thermogenic organ that contains a high amount of mitochondria with uncoupling protein 1 (UCP1). BAT is able to metabolize primarily free fatty acids (FFA) but also glucose, amino acids, and ketones in order to produce heat through UCP1 [5]. Through increased activity of the sympathetic nervous system (SNS) and the corresponding release of norepinephrine (NE), cold is the most potent physiological activator of BAT [6,7].

The presence of BAT in infants has been known for decades whereas the continued existence of BAT in adults has been questioned [8]. In 2009 findings using 2-deoxy-2-(^18^F)fluoro-d-glucose positron emission tomography (FDG PET) verified the presence of metabolically active BAT in adult humans [9,10,11] and since then BAT has been revitalized as a potential target organ in the treatment of obesity and metabolic syndrome in humans [7,12]. BAT in rodents has been extensively investigated but knowledge transfer from rodents to humans has proven difficult [13].

Currently, the gold standard for BAT imaging is FDG PET/Magnetic Resonance Imaging (MRI) in rodents [14] or FDG PET/CT in humans [15,16]. These well-known imaging modalities rely on the radioactive tracer ^18^FDG in the detection of tissues with high glucose consumption, for example, activated BAT. The FDG signal gives an indication of the distribution of glucose uptake and phosphorylation in the tissue. Other imaging modalities have been used for BAT assessment such as infrared thermography. However, due to subcutaneous fat thickness, fur/hair, camera angle influences, and so on, measurement of BAT activity has been shown to be very difficult using this method [17,18].

Finally, a promising method to detect BAT activity is the [1-^13^C]labelled hyperpolarized MRI method (HP-MRI). This method relies on hyperpolarized metabolic non-radioactive bio-probes allowing studies of metabolic processes under real time conditions in vivo [19,20,21,22,23,24]. Hyperpolarization by the dynamic-nuclear-polarization (DNP) method gives the advantage of a 10.000-fold increased signal compared to the thermal signal at room temperature at clinical (MRI) magnetic field strengths [25]. When a hyperpolarized metabolite such as [1-^13^C]pyruvate is infused into the circulation, the signal is traced to the downstream metabolic products of pyruvate (difference in MRI signal signature). The procedure enables real-time in vivo visualization of normal and abnormal metabolism, for example, changes in the balance between aerobic and anaerobic metabolism or changes in substrate utilization. As it is an MRI-based technique and no radioactive solutions are involved, this method may also open up to safe repetitive investigations in humans.

In BAT, SNS activation upon cold exposure increases the overall metabolism through UCP1 activation yielding a higher activity of the pyruvate dehydrogenase enzyme complex. A recent proof-of-concept study in rats has used HP-MRI to visualize active BAT [22]. A correlation between BAT activation, via injected NE and increased signal amplitude of the hyperpolarized [1-^13^C]pyruvate metabolites, was demonstrated. However, in this latter study the HP-MRI measurements were not compared to the conventional FDG PET scans. Moreover, no determination of UCP1 protein was performed in this study. Thus, HP-MRI may add additional metabolic information compared to the conventional FDG PET-based method. Moreover, the method gives the advantage for successive imaging given the short half-life of the hyperpolarized bio-probes and the fact that it is a non-radiation based technique. However, before applying this method in humans, proof-of-concept studies in well-known animal models are warranted.

The aim of this study was to determine if the HP-MRI method could be used for in vivo assessments of BAT activity in mice in which BAT activity was stimulated by long-term cold exposure as compared to thermo-neutrality. We hypothesized that cold exposure increases BAT activity and thereby the overall uptake and metabolism in BAT, as measured by increase in all downstream metabolites from [1-^13^C]pyruvate such as bicarbonate, lactate, and alanine using HP-MRI, yielding increases in [1-^13^C]metabolite/[1-^13^C]pyruvate ratios. Moreover, *UCP1*gene expression and protein measurements in interscapular BAT (iBAT) were assessed and FDG PET/MRI was performed for comparative analysis.

## 2. Results

### 2.1. UCP1 Levels in iBAT

**Figure 1:**
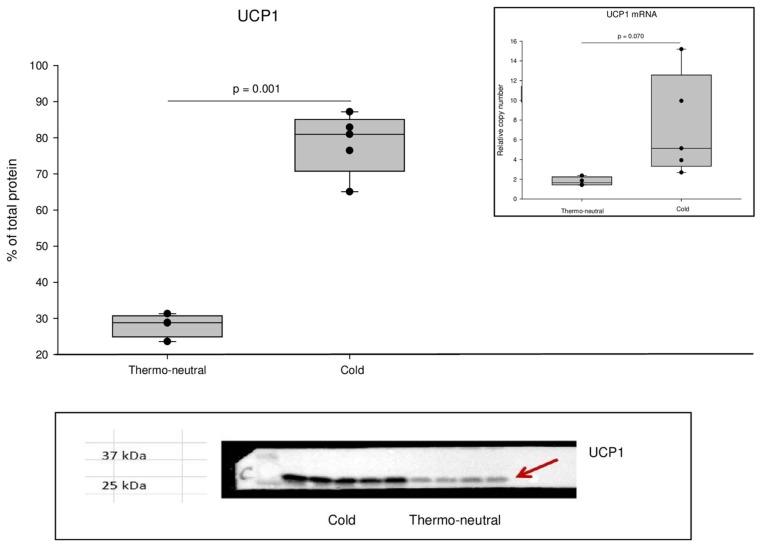
UCP1 protein and *UCP1*mRNA expression in interscapular brown adipose tissue (iBAT) biopsies. UCP1 protein levels were determined by Western blot (a representative Western blot is shown in Inset lower panel) and *UCP1*mRNA expression was determined by qPCR (Inset upper panel). For Western blot: *n* = 5 (cold) and *n* = 4 (thermo-neutral) and for qPCR: *n* = 5 (cold) and *n* = 4 (thermo-neutral).

UCP1 was significantly increased after cold exposure compared to thermo-neutral conditions with a 3-fold increase in protein levels (*p* = 0.001) and a 4-fold increase in mRNA expression (*p* = 0.07; Figure 1).

### 2.2. HP-MRI Determination of iBAT Activity

**Figure 2:**
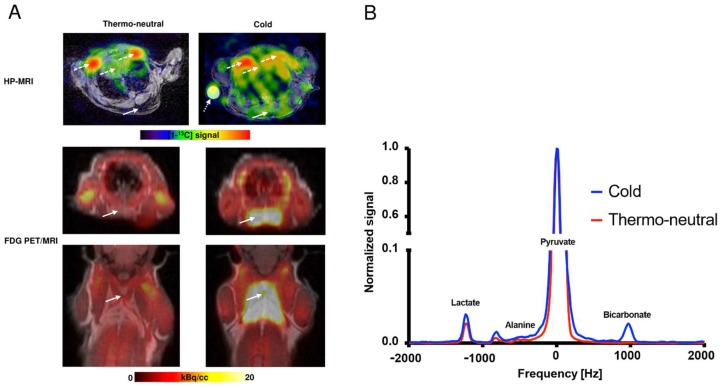
Visualization of iBAT using hyperpolarized MRI (HP-MRI) and FDG PET-MRI. In (**A**) iBAT in a mouse under thermo-neutral and a mouse under cold conditions visualized by HP-MRI (transaxial projection) or FDG PET/MRI (transaxial and coronal projection) are shown (400× magnification). The solid arrow demonstrates the iBAT region and dashed arrows demonstrates the heart and large vessels (subclavian artery). The dotted arrow in the cold HP-MRI image represents a phantom. The color scale indicates [1-^13^C]signal intensity or FDG uptake using either HP-MRI or PET/MRI, respectively. The analysis was performed on spectra only from the regions of interest (ROI) drawn on the iBAT tissue. Due to slice thickness, signal from the heart and large vessels (subclavian artery) is seen in both thermo-neutral and cold animals. In (**B**) two representative iBAT [1-^13^C]spectra from a cold acclimatized (blue) and a thermo-neutral (red) mouse are shown. The [1-^13^C]signal is normalized to [1-^13^C]pyruvate in iBAT and frequency is measured in Hz.

The HP-MRI was performed immediately after injection of hyperpolarized [1-^13^C]pyruvate. This enabled the production of an image of metabolic activity in the iBAT which was compared to the FGD PET uptake as shown in Figure 2A. Two representative [1-^13^C]spectra from cold (blue) and thermo-neutral (red) mice are shown in Figure 2B.

**Figure 3:**
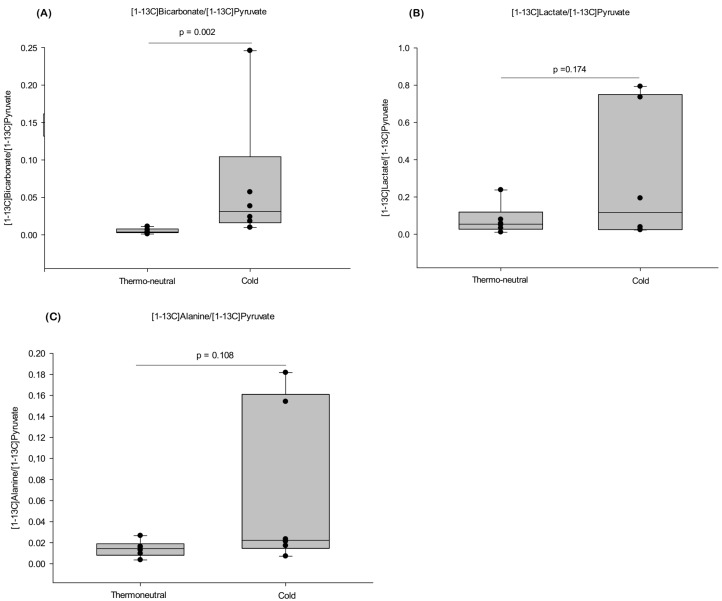
Effect of cold versus thermo-neutrality on iBAT activity determined by HP-MRI. [1-^13^C]signal intensity of HP-MRI showing metabolites normalized to maximum [1-^13^C]pyruvate signal in iBAT during cold exposure and thermo-neutral conditions, respectively. In (**A**) the [1-^13^C]bicarbonate/[1-^13^C]pyruvate ratio is shown, (**B**) [1-^13^C]lactate/[1-^13^C]pyruvate ratio and (**C**) [1-^13^C]alanine/[1-^13^C]pyruvate ratio. *n* = 6 in each of the two groups. *p*-values are related to the comparison between thermo-neutral and cold group.

After injection of [1-^13^C]pyruvate the [1-^13^C]signal from the downstream metabolites of pyruvate (bicarbonate, lactate, and alanine) were normalized to the maximum [1-^13^C]pyruvate signal obtained in iBAT (Figure 3).

Injection of NE did not affect pyruvate metabolism in neither the cold exposed mice nor in the thermo-neutral mice and these two groups (+/− NE treated) were therefore pooled resulting in six mice in both the cold exposed group and the thermo-neutral group (Figure S1).

After cold exposure the [1-^13^C]bicarbonate/[1-^13^C]pyruvate signal was significantly increased by 13-fold (*p* = 0.002) as compared to the thermo-neutral conditions (Figure 3A). Moreover, the [1-^13^C]lactate/[1-^13^C]pyruvate and [1-^13^C]alanine/[1-^13^C]pyruvate ratio also increased by 4- and 5-fold, respectively, after cold exposure (Figure 3B,C).

**Figure 4:**
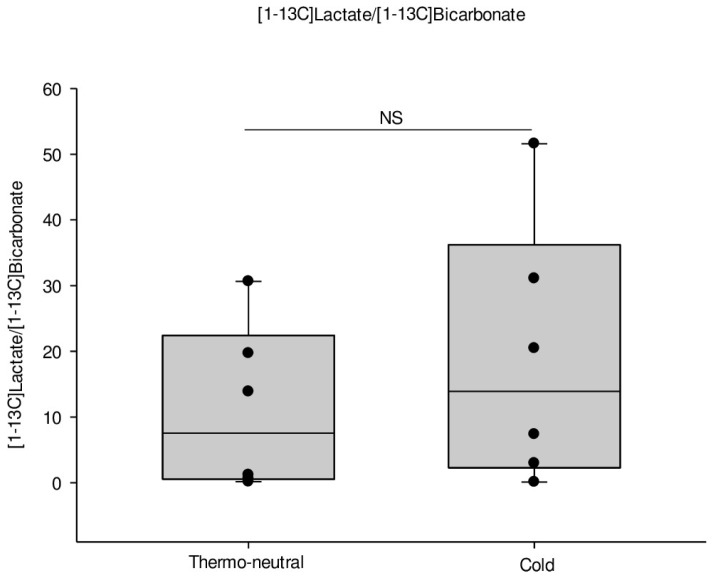
[1-^13^C]lactate/[1-^13^C]bicarbonate ratio corresponding to the ratio between anaerobic and aerobic metabolism in iBAT determined by HP-MRI. An increased [1-^13^C]lactate/[1-^13^C]bicarbonate ratio in the cold acclimatized mice by a 1.7-fold increment compared to thermo-neutral mice was observed, but this difference was not statistically significant (*p* = 0.429). *n* = 6 in each group.

The balance between the anaerobic and aerobic metabolism can be found by the [1-^13^C]lactate/[1-^13^C]bicarbonate ratio as previously described in rat heart [26]. There was a 1.7-fold increased ratio in the cold acclimatized mice (Figure 4).

Neither the [1-^13^C]alanine/[1-^13^C]lactate ratio nor the [1-^13^C]bicarbonate/[1-^13^C]alanine ratio differed between cold acclimatized and thermo-neutral mice (data not shown.

**Figure 5:**
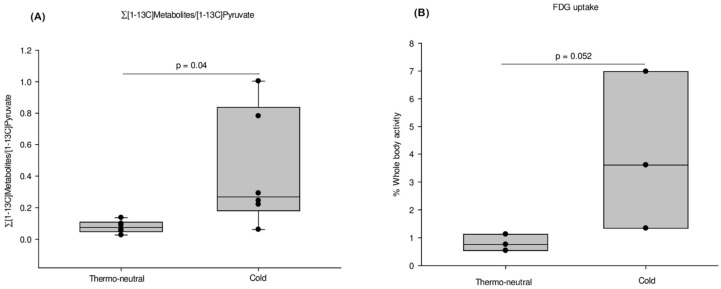
The FDG PET/MRI and HP-MRI methods both imaging iBAT activity. The sum of [1-^13^C]-metabolites (∑[1-^13^C]-metabolites/ [1-^13^C]pyruvate) using the HP-MRI method is shown in (**A**). The signal of FDG PET/MRI is presented as percentage FDG activity in iBATcompared to whole body activity in a voxel of interest (VOI) marked (**B**). Cold exposure is compared to thermo-neutrality. For both methods; *n* = 3 in FDG PET/MRI study and *n* = 6 in the HP-MRI study.

The total sum of metabolized pyruvate (∑[1-^13^C]-metabolites/[1-^13^C]pyruvate ratio) was increased 5.5-fold increment in the cold acclimated group compared to the thermo-neutral group (Figure 5A).

### 2.3. FDG PET/MRI Determination of iBAT Activity

The iBAT activity measured by FDG PET/MRI was determined in another group of cold acclimatized and thermo-neutral mice (Figure 5B). The FDG uptake was increased 5-fold in the cold acclimatized mice (3.977 ± 2.838% of whole body activity) as compared to the thermo-neutral mice (0.808 ± 0.296% of whole body activity) (Figure 5B, *n* = 3 in both groups).

## 3. Discussion

By direct investigation of iBAT, cold exposure increased, as expected, both *UCP1*gene expression and UCP1 protein by 3–4 fold indicating that cold exposure increased thermogenesis in the mice. Furthermore, the present study demonstrates that the HP-MRI method could be used to determine both non-stimulated as well as stimulated iBAT activity in mice. Moreover, cold exposure as compared with thermo-neutrality was found to increase the activity of iBAT by 5.5-fold increase in pyruvate metabolism which is in good agreement with the 5-fold increment in FDG uptake in iBAT by the FDG PET/MRI method. Thus, overall there was a good agreement in the cold-induced activation of iBAT using various methods in the present study.

All three metabolites of the hyperpolarized pyruvate ([1-^13^C]bicarbonate, [1-^13^C]lactate, and [1-^13^C]alanine) were increased 4-fold or more adjusted for total [1-^13^C]pyruvate after cold exposure. This indicates a generally increased carbohydrate metabolism within iBAT, which could originate from either increased substrate entering the cells and/or increased intracellular enzyme concentration/activity [27]. In order to determine whether cold induces differential effects on the anaerobic versus the aerobic pathways (independent of perfusion and uptake), we determined the [1-^13^C]lactate/[1-^13^C]bicarbonate ratio and found that this ratio was increased 1.7-fold by cold exposure. Thus, this result shows a tendency towards increased anaerobic metabolism during cold exposure as shown in other studies [5,22]. Thus, cold exposure may primarily upregulate the pyruvate dehydrogenase enzyme complex, but the tricarboxylic acid (TCA) flux, lactate dehydrogenase, and alanine aminotransferase may be affected as well. However, the ultimate mechanism for this upregulation may be the cold-induced upregulation of UCP1 with enhanced use of glucose and FFA for heat production. This enhanced metabolism in BAT (e.g., enhanced oxygen demand, enhanced heat production etc.) will result in increased blood flow to BAT and hence enhanced delivery of [1-^13^C]pyruvate to the BAT cells during the HP-MRI procedure. Our results from the [1-^13^C]metabolites are adjusted to the total amount of [1-^13^C]pyruvate in iBAT and, therefore, the results are already adjusted for differences in blood flow in iBAT, so our results indicate an increase in metabolic activity independent of changes in blood flow. Moreover, we rely on the metabolic derivative ratio ([1-^13^C]alanine/[1-^13^C]lactate, [1-^13^C]bicarbonate/[1-^13^C]alanine and l[1-^13^C]actate/[1-^13^C]bicarbonate) as perfusion/uptake insensitive measures of the metabolic phenotype. This has been demonstrated to be a particularly useful measure in situations with uncertain perfusion/uptake conditions, as pyruvate is largely found in the vascular bed and the derivatives are largely produced intracellularly. Thus, the ratio between two formed metabolic products are less affected by perfusion and uptake than the pyruvate normalization [28].

Lau et al. [22] have published the only other study on iBAT using the hyperpolarized [1-^13^C]pyruvate MRI method. They found that activation of iBAT with NE in rats resulted in a 3.9-fold increment of [1-^13^C]lactate and a 6.3-fold increment of [1-^13^C]bicarbonate after injection of hyperpolarized [1-^13^C]pyruvate. Basically, our study confirmed the findings of Lau et al. [22] that the HP-MRImethod can detect BAT activity. Our study adds, however, to the study of Lau et al. by showing that the stimulation of iBAT (fold increment) detected by HP-MRI was very similar to other methods/ways to determine activated BAT such as FDG PET scan and biological methods as gene expression and protein determination of UCP1 emphasizing the usefulness of the HP-MRI method. We were, however, not able to detect any effect after NE injection in our HP-MRI study. There are, however, several differences between the two studies: for example, Lau et al. [22] used rats and we used mice, their study was performed at room temperature (22 °C) while ours were performed at thermo-neutrality (30 °C) or long term cold exposure (6 °C). Another reason for the different effect of NE may be that different anesthetics were used in the two studies. Lau et al. [22] used ketamine/xylazine injections, whereas we used isoflurane and sevoflurane. It has been shown mostly in vitro [29] but also in vivo [30] that these gasses may inhibit NE-induced BAT activity. We believe that the two imaging methods support the conclusion of iBAT activation in cold mice even when using volatile anesthetics. That acute injection of NE determined by the HP-MRI method neither affected the iBAT activity at thermoneutrality nor after cold exposure supports the notion that the volatilegasses may inhibit this acute effects of NE. However, on the other hand they do not affect the effect of long standing cold exposure possibly due to an increment of the BAT organ during this condition. Thus, the choice of anesthetics, preferably selecting anesthetics without effects on BAT activity should be considered carefully in future animal studies focusing on BAT activity.

The relatively small sample size may pose limitations to the study. However, the robust 5-fold difference between thermo-neutrality and cold exposure found by the new method is very similar to our findings using the well-established FDG PET method and fitted well with the observed increase in *UCP1*mRNA and protein levels. Therefore, we are confident with our data and find it unlikely that a bigger sample size would change our conclusions.

The [1-^13^C]pyruvate HP-MRI modality might be further improved by using more specific MRI acquisitions such as quantification of BAT using, for example, fat fraction of BAT [31], assessment of blood flow [32], or oxygen extraction [33]. All these measures may provide further information on BAT metabolism in both rodents and humans. In this study, field of view (FOV) in HP-MRI was somewhat lower compared to FDG PET/MRI but the technique itself does not limit FOV and as such full body examinations in humans are feasible. However, this requires more advanced MRI sequences than used in this study (small animal system).

Translation of the HP-MRI method for BAT measurements into human studies poses some considerations. So far, three human studies using HP-MRI have been published [24,33,34] where HP-MRI was used for metabolic imaging of organs such as the prostate (cancer detection and treatment evaluation) and the heart, thereby showing the feasibility of application of such methodology in tissues with high metabolic rates in humans. Detection sensitivity may, however, very well be different for human BAT. However, the HP-MRI method, by being radiation free and non-invasive, may turn out to be an important investigational tool to assess the efficacy of different drugs and/or substances that aim at increasing BAT activity.

It has been shown that FDG uptake varies greatly in humans, with uptake values that depend on gender, age, and BMI. Pyruvate uptake and metabolism may be expected to vary in a similar way. However, because pyruvate uptake is dependent on the monocarboxylate transporter 1 and 4 (MCT1 and MCT4) [35,36] whereas FDG uptake is dependent on the glucose transporter 1 and/or 4 (GLUT1 and/or GLUT4), the latter being insulin dependent, differences may be seen in the uptake of these two substrates.

Moreover, presently there are limited sites around the world that performs HP-MRI and as such this is a bottle neck for its clinical use. Finally, we believe that the relatively high costs of hyperpolarization solutions will drop as the HP-MRI method emerges into clinical use.

## 4. Materials and Methods

### 4.1. Animal Handling

Seven-week old male C57BL/6 mice (*n* = 18, weight = 24.9 ± 1.7 g) were housed in groups of three per cage in a climate controlled chamber (HPP750life Memmert GmbH + Co. KG, Schwabach, Germany). A 12 h light:dark cycle (6 a.m.–6 p.m.) was maintained throughout the housing period and the mice had access to *ad libitum* standard chow and water. The mice were purchased in two separate batches from the same vendor (Janvier labs, Le Genest-Saint-Isle, France). The Danish Expectorate of Animal Experiments approved all animal experiments (project identification code 2015-15-0201-00709, date 07-12-2015).

### 4.2. Protocol for Temperature Acclimatization

After five days of acclimatization at room temperature the mice were randomly subjected to a long-term acclimatization protocol; either a thermo-neutral protocol (*n* = 9) or a cooling protocol (*n* = 9). Mice subjected to the thermo-neutral protocol were kept at 30 °C for 3–4 weeks after which they were scanned with either HP-MRI or FDG-PET/MRI. The mice subjected to the cooling protocol were gradually cooled over a ten-day period from 22 to 6 °C and then maintained at 6 °C for 2–3 weeks (in total 3–4 weeks of cold acclimatization) until they were scanned with either HP-MRI or FDG PET/MRI. Humidity was maintained at 40% during both interventions. The mice were monitored daily to assess general well-being and no mice died during these treatments. The scanning procedures for both the HP-MRI and FDG PET scans were performed at room temperature.

### 4.3. HP [1-^13^C]pyruvate-MRI

Non-fasted mice (6 cold acclimated and 6 thermo-neutral mice) were anesthetized by sevoflurane after removal from the climate chamber. An intravenous catheter was inserted in a tail vein. Subsequently, the mice were positioned in the scanner in a supine position. The first intravenous hyperpolarized [1-^13^C]pyruvate injection was administered over a ten second period followed by the first HP-MRI as previously described [32,37]. The MRI was performed at room temperature.

In order to obtain maximal stimulation of BAT, half of the mice were injected with 2.5 mg/kg NE intraperitoneal (i.p.) after a one-hour washout-period, the other half of the mice were injected with saline. After further 15 min a second hyperpolarized [1-^13^C]pyruvate injection was administered and a second MRI was performed. Body temperature was maintained using an air heater and core temperature was monitored using a rectal thermometer. Respiration rate and heart rate were monitored throughout the scans.

The hyperpolarization protocol was as follows: a solution of 127 mg of [1-^13^C]pyruvic acid (Cambridge Isotope Laboratories, Tewksbury, MA, United States of America (USA)) was mixed with 15 mM AH111501 (GE Healthcare, Broendby, Denmark) and polarized in a 5T SPINlab (GE Healthcare, Broendby, Denmark). This protocol resulted in a reproducible polarization of 40% with a final concentration of 125 mM. Image acquisition was performed 15 s after injection of hyperpolarized [1-^13^C]pyruvate [37]. Standard ^1^H and hyperpolarized ^13^C were performed on a 9.4T pre-clinical MRI system (Agilent, Santa Clara, CA, USA) equipped with a dual tuned ^1^H/^13^C volume transmit (Doty scientific, Columbia, SC, USA). The 15 s delay was automatically inserted at the start of the injection to reduce the variability, similar to previous publications [20,38,39]. The software package vnmrJ 4.0 (Agilent, Santa Clara, CA, USA) was used for acquisition and reconstruction. Prior to the T_1_ weighted image, a ^1^H sequence was used to position the ^13^C slice. A slice-selective 2D ^13^C chemical-shift imaging sequence was used for hyperpolarized [1-^13^C]pyruvate imaging. The resolution obtainable with hyperpolarized MRI is largely dependent on the choice of MRI sequence. In this study a robust chemical shift imaging (CSI) sequence was chosen with less than 2 mm in plane resolution and maximum intensity projection (MIP) slab of 10 mm. However, more advanced sequences have been demonstrated to allow faster and higher resolution and thus improvement beyond PET resolution both temporal and spatial is feasible especially in humans due to the size constraints associated with the imaging of small animals [33,39,40,41,42].

Parameters were: flip angle = 10º, image matrix of 16 × 16, repetition time/echo time (TR/TE) = 65 ms/0.69 ms, FOV = 30 × 30 mm (size of each pixel was 1.875 mm × 1.875 mm), a slice thickness of 10 mm, spectral width = 8012 Hz, data sampling = 512 complex points. Data analysis of ^13^C MRI data was performed in VnmrJ 4.0 and transferred to MATLAB (R2016a).

Interscapular BAT depots (iBAT) were identified and regions of interest (ROIs) were manually drawn on the proton MR images by a blinded investigator. The ROIs were transferred to the hyperpolarized images and spectra of the different metabolites were obtained. Subsequently, the different metabolic compounds were normalized to the maximum [1-^13^C]pyruvate signal in iBAT. Cold acclimated mice were compared with thermo-neutral mice as an assessment of metabolic activity in iBAT.

### 4.4. FDG PET/MRI

The scan was performed on a Mediso nanoScan PET/MRI (Mediso, Budapest, Hungary). The non-fasted mice (3 cold acclimated and 3 thermo-neutral mice) were anesthetized by isoflurane and received a NE injection (2.5 mg/kg) i.p. for maximal activation of BAT. After 15 min FDG (15 ± 8 mBq) was injected in a tail vein and a 30 min emission recording initiated 60 min post-injection [43,44]. A T1-weighted MRI sequence was acquired for anatomical reference. Scan positioning and monitoring during the procedures were equivalent to the HP-MRI protocol.

For assessment of the FDG PET scans the iBAT depots were identified on MRI and voxel of interest (VOIs) manually drawn on the most FDG avid voxels.

The signal intensity for each VOI was calculated as percent FDG activity normalized to whole body activity in a VOI.

HP-MR scans and FDG-PET scans were performed at two separate departments using two different but similar gas anesthetics.

### 4.5. Gene Expression and Protein Determination

Following both HP-MRI and FDG PET/MRI the mice were sacrificed by cervical dislocation, the iBAT region was identified and dissected. The tissue was weighed and snap-frozen in liquid nitrogen and stored at −80 °C.

*UCP1*gene expression and UCP1 protein were measured in iBAT. Tissue samples from cold acclimatized mice (*n* = 5) and thermo-neutral mice (*n* = 4) were evaluated by qPCR in a LightCycler 480 (Roche, Penzberg, Germany) as previously described [45]. Data are shown as relative copy number compared to the housekeeping gene Peptidylpropyl Isomerase A (PPIA). The following primer sequences designed using QuantPrime software [46] were PPIA (forward 5′-TCCTGGCATCTTGTCCAT-3′ reverse 5′TGCTGGTCTTGCCATTCCT-3′, product length = 179 bp) and UCP1 (forward 5′-GCCATCTGCATGGGATCAAACC-3′ reverse 5′-TCGTCCCTTTCCAAAGTGTTGAC-3′, product length = 99 bp).

Western Blot analysis was performed in iBAT samples from five cold acclimatized mice and four thermo-neutral mice using a UCP1 primary antibody (Cat#ab10983, Abcam, Cambridge, UK) as previously described [47]. UCP1 protein levels were normalized to the total amount of protein in the sample.

### 4.6. Statistical Method

All data are presented as mean ± SEM and analysis was performed using Sigma Plot (Version 12.5, Systat, San Jose, CA, USA). Differences between groups were calculated using Students *t*-test on normally distributed data. Log-transformation was performed when necessary to achieve Gaussian distribution. Differences in each temperature group after NE injection were tested using One-way repeated measures ANOVA. Primer stability was tested using one-way ANOVA. Statistical significance was achieved at *p* ≤ 0.05.

## 5. Conclusions

This study demonstrates the detection of iBAT by the novel HP-MRI imaging modality comparable to the well-established FDG PET/MRI technique. The HP-MRI method allows a deeper insight of the oxidative pathways involved during BAT activation, for example, aerobic versus anaerobic pathways and may therefore add important information to the conventional FDG PET/CT method in future human BAT research. Furthermore, this method may open up for repetitive physiological investigations of BAT also in young humans because it is a non-radiation based technique. 

## Supplementary Materials

Supplementary materials can be found at http://www.mdpi.com/1422-0067/19/9/2597/s1.
