# Supplementary material for: Evaluation of Active Brown Adipose Tissue by the Use of Hyperpolarized [1-13C]Pyruvate MRI in Mice
Source: Int J Mol Sci. 2018 Sep 1;19(9):2597. doi: 10.3390/ijms19092597 (PMC6164296; doi:10.3390/ijms19092597)
Supplement: Supplementary file 1 [file ijms-19-02597-s001.zip › ijms-333483-figures and supplemenary-proofreading/ijms-333483-supplementary.docx]

**Figure S1.** Effect of norepinephrine injection in cold exposed and thermo-neutral mice. In (**A**) the [1-^13^C]bicarbonate/[1-^13^C]pyruvate ratio is shown, (**B**) [1-^13^C]lactate/[1-^13^C]pyruvate ratio and (**C**) [1-^13^C]alanine/[1-^13^C]pyruvate ratio. *n* = 6 in the non-treated and *n* = 3 in the NE-treated groups. There was no significant difference between +/− NE.
