# Supplementary figures and images for: Evaluation of Active Brown Adipose Tissue by the Use of Hyperpolarized [1-13C]Pyruvate MRI in Mice
Source: Int J Mol Sci. 2018 Sep 1;19(9):2597. doi: 10.3390/ijms19092597 (PMC6164296; doi:10.3390/ijms19092597)

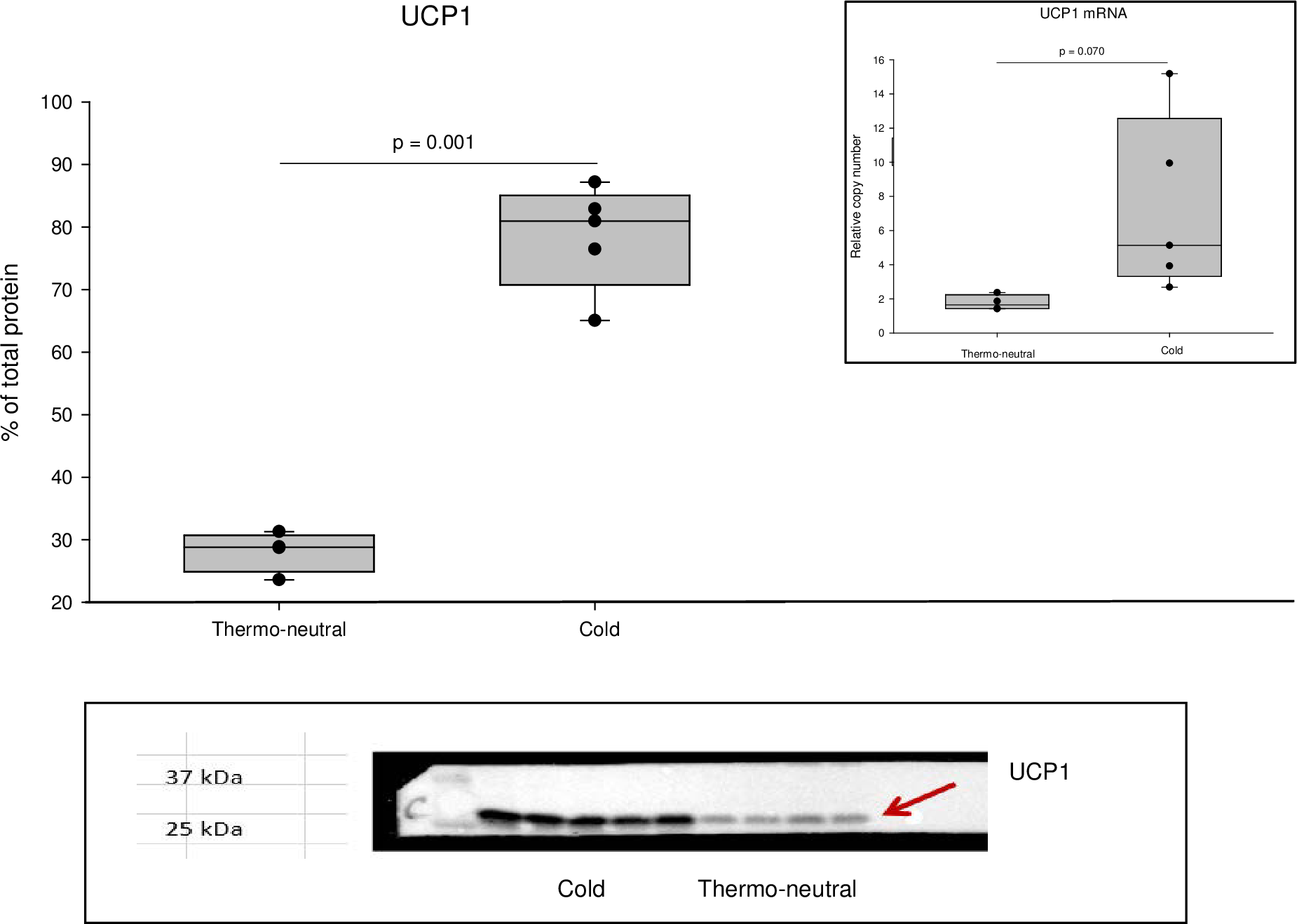

Supplement: Supplementary file 1 [file ijms-19-02597-s001.zip › ijms-333483-figures and supplemenary-proofreading/Fig1.tiff]

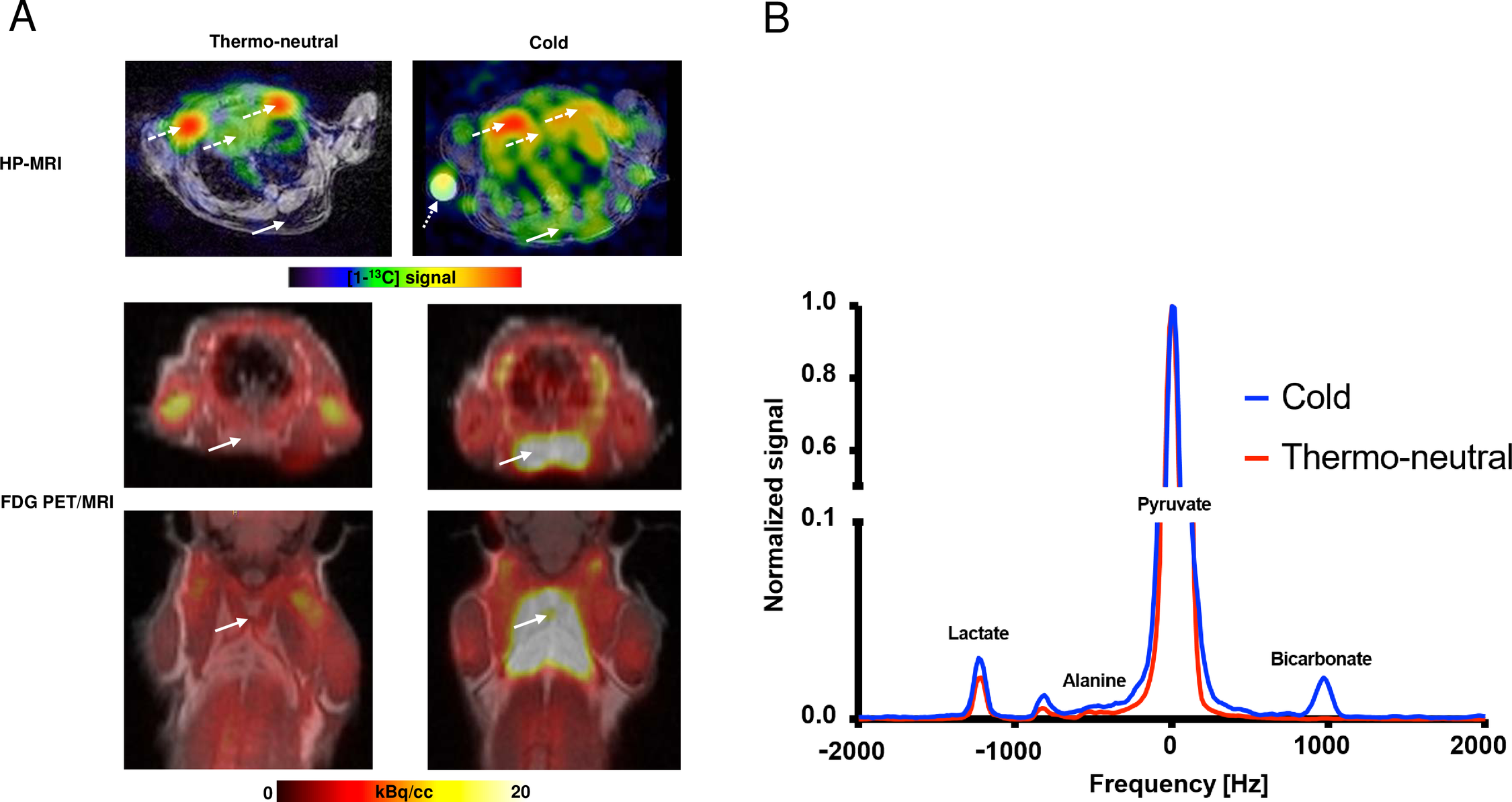

Supplement: Supplementary file 1 [file ijms-19-02597-s001.zip › ijms-333483-figures and supplemenary-proofreading/Fig2.tiff]

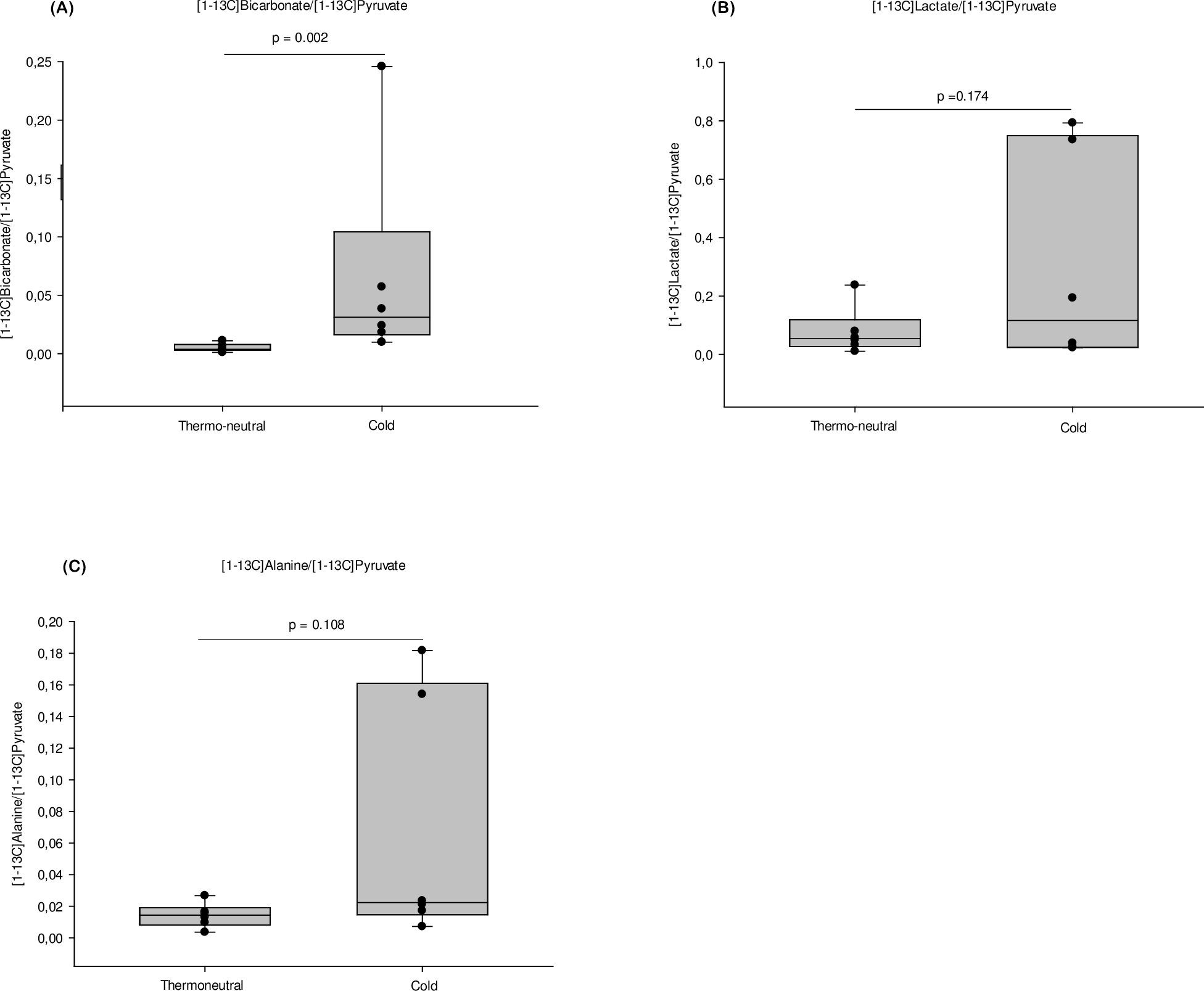

Supplement: Supplementary file 1 [file ijms-19-02597-s001.zip › ijms-333483-figures and supplemenary-proofreading/Fig3.tiff]

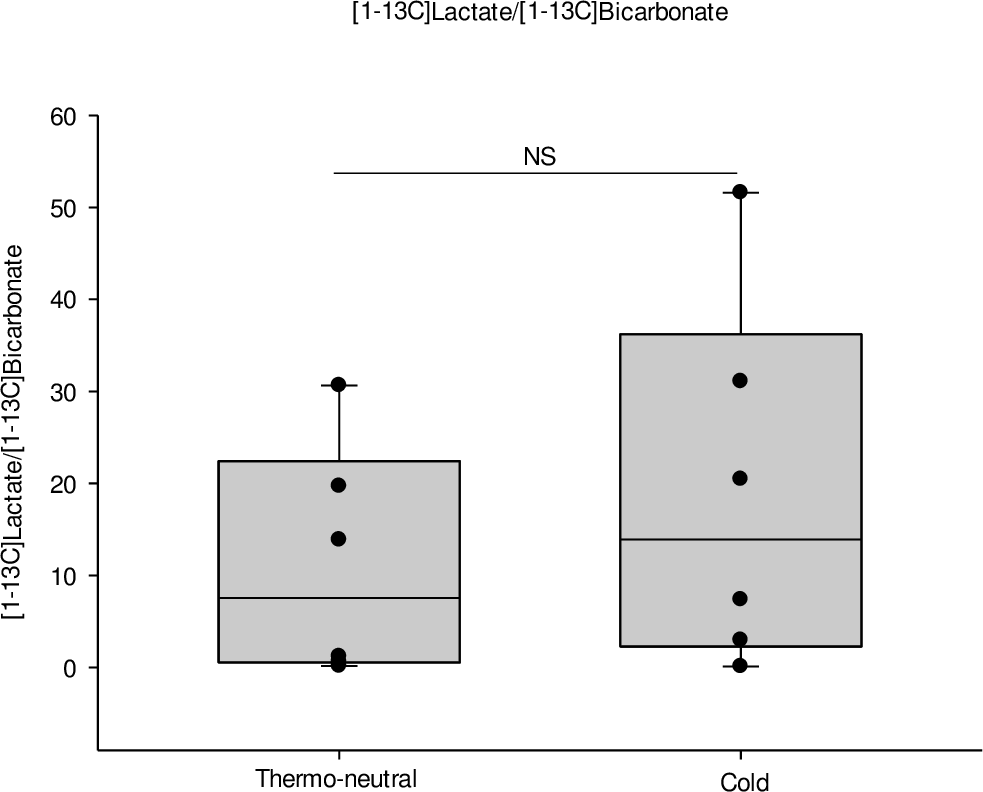

Supplement: Supplementary file 1 [file ijms-19-02597-s001.zip › ijms-333483-figures and supplemenary-proofreading/Fig4.tif]

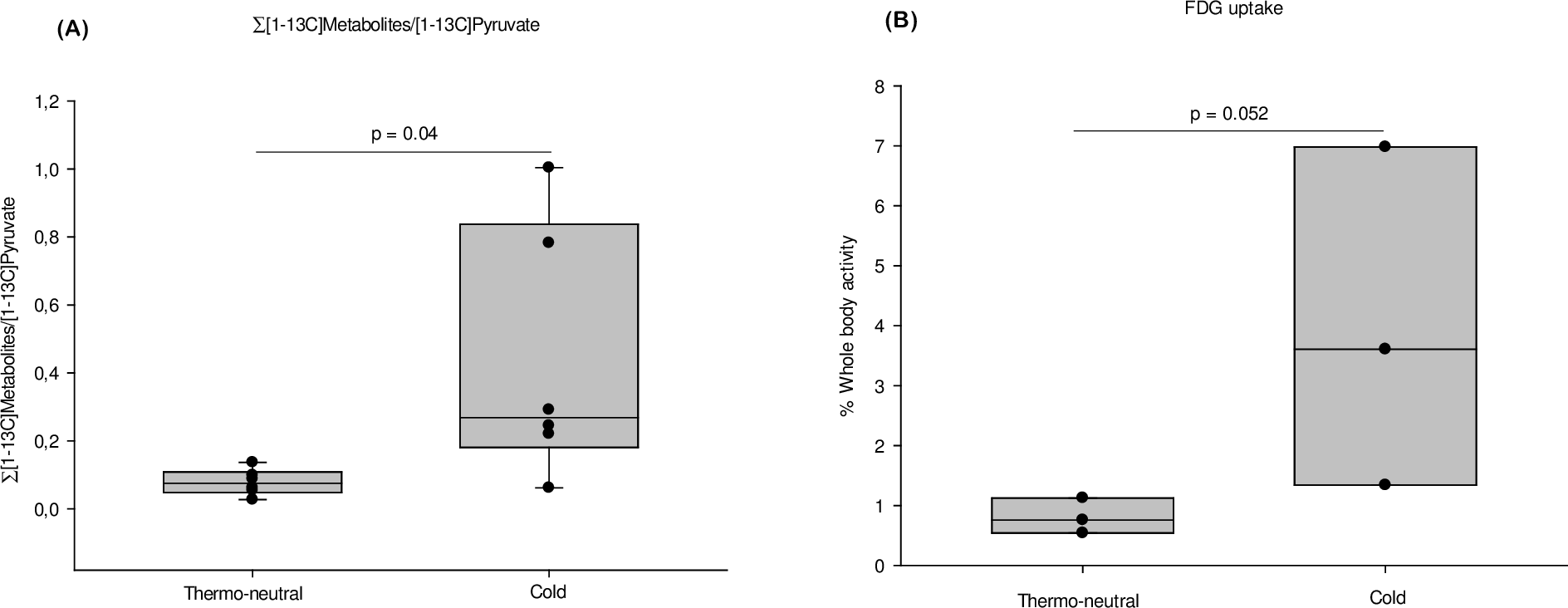

Supplement: Supplementary file 1 [file ijms-19-02597-s001.zip › ijms-333483-figures and supplemenary-proofreading/Fig5.tiff]
